# Phase 2 Prospective Trial of Retreatment with [^177^Lu]Lu-PSMA-617 Molecular Radiotherapy for Metastatic Castration-Resistant Prostate Cancer—RE-LuPSMA

**DOI:** 10.2967/jnumed.125.271231

**Published:** 2026-05

**Authors:** John Nikitas, Adrien Holzgreve, Jesus Juarez, Koichiro Kimura, Stephanie Lira, Hamzah Alam, Maria Contreras, Lela Theus, Andrew T. Nguyen, Zachary Ells, Shaojun Zhu, Tristan Grogan, David A. Elashoff, Lena M. Unterrainer, Magnus Dahlbom, Martin Allen-Auerbach, Johannes Czernin, Jeremie Calais

**Affiliations:** 1 Department of Radiation Oncology, University of Pennsylvania, Philadelphia, Pennsylvania;; 2 Ahmanson Translational Theranostics Division, UCLA, Los Angeles, California;; 3 Department of Nuclear Medicine, LMU University Hospital, LMU Munich, Munich, Germany; and; 4 Department of Medicine Statistics Core, UCLA, Los Angeles, California

**Keywords:** metastatic castration-resistant prostate cancer, mCRPC, [^177^Lu]Lu-PSMA-617, radiopharmaceutical therapy, prostate-specific membrane antigen, PSMA

## Abstract

[^177^Lu]Lu-PSMA-617 radiopharmaceutical therapy has been approved for the treatment of patients with metastatic castration-resistant prostate cancer for up to 6 cycles. Unfortunately, this treatment is not curative and patients experience relapse, even after initially favorable responses. When this occurs, patients have limited treatment options. Readministration of [^177^Lu]Lu-PSMA-617 in patients who previously benefited from therapy and had limited toxicity seems to be a promising option, with retrospective studies reporting favorable outcomes. **Methods:** RE-LuPSMA is an investigator-initiated, single-arm, single-center, open-label, phase 2 clinical trial designed to study the efficacy and safety of rechallenge therapy using [^177^Lu]Lu-PSMA-617 in patients whose disease progressed after responding well to a previous regimen of [^177^Lu]Lu-PSMA-617. This study plans to enroll 40 patients with progressive metastatic castration-resistant prostate cancer who previously completed 4–6 cycles of [^177^Lu]Lu-PSMA-617 with a favorable response (i.e., ≥50% decrease in prostate-specific antigen [PSA] level at any point during the first [^177^Lu]Lu-PSMA-617 regimen). After the first regimen of [^177^Lu]Lu-PSMA-617, patients must meet VISION trial criteria on a prostate-specific membrane antigen (PSMA) PET/CT scan within 8 wk of the planned first cycle of rechallenge therapy. After enrollment, participants will receive up to 6 additional cycles of [^177^Lu]Lu-PSMA-617 (7.4 GBq every 6 wk). The primary endpoint is 12-mo overall survival (OS), measured from the start of rechallenge therapy. The study will have 80% power to detect a difference between the null hypothesis of 50% OS at 12 mo and the study hypothesis of 71% OS at 12 mo. Secondary endpoints include adverse-event rates, PSA response rates (proportion of patients with a decrease of 50% or greater in PSA level), biochemical progression-free survival (defined as the time until PSA level increases 25% and 2 ng/mL above the nadir), radiographic progression-free survival, and quality-of-life changes (measured using Functional Assessment of Cancer Therapy–Radionuclide Therapy and Brief Pain Inventory–Short Form). Enrollment began in August 2024, with a planned study duration of 45 mo.

## Visual Abstract

**Figure:**
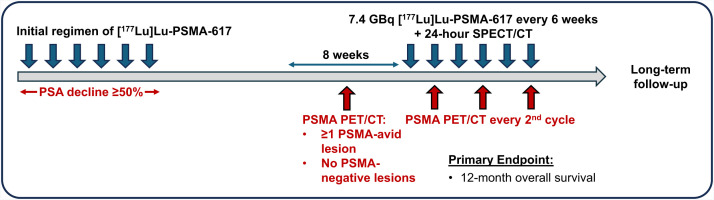

The phase 3 VISION trial demonstrated that [^177^Lu]Lu-PSMA-617 radiopharmaceutical therapy (RPT) can improve overall survival (OS), radiographic progression-free survival (PFS), prostate-specific antigen (PSA) response, quality-of-life scores, and bone metastasis pain compared with standard of care alone in patients with progressing metastatic castration-resistant prostate cancer (mCRPC) who previously received 1 or 2 taxane-based chemotherapy regimens and at least 1 androgen receptor pathway inhibitor (ARPI) (1,2). The phase 3 TheraP trial found that, for patients with mCRPC for whom cabazitaxel was considered the next line of therapy, [^177^Lu]Lu-PSMA-617 RPT evoked a superior PSA response and fewer severe adverse effects compared with cabazitaxel (3).

In 2022, the Food and Drug Administration (FDA) and European Medicines Agency approved the use of [^177^Lu]Lu-PSMA-617 RPT for the treatment of patients with mCRPC who had disease progression after chemotherapy (4). The approved regimen consists of 6 cycles of treatment, approximately 6 wk apart, with one 7.4-GBq injection per cycle (5).

Unfortunately, this treatment is not curative, and patients experience relapse, even after initially favorable responses. When this occurs, patients have limited treatment options, given that they previously received taxane-based chemotherapy or ARPI regimens. Readministration of [^177^Lu]Lu-PSMA-617 seems to be a promising option for patients who responded well to treatment and experienced limited toxicity.

Rechallenge therapy, in which [^177^Lu]Lu-PSMA-617 is reintroduced to patients whose disease progresses after a treatment completion, has been reported in multiple small retrospective series (6–10). Overall, these studies have reported favorable PSA response rates, with 37%–73% of patients experiencing PSA declines of 50% or greater with rechallenge therapy (6–10). They also reported predominantly hematologic adverse events with minimal kidney toxicity. Furthermore, in the series reported by Violet et al., the 15 patients who received additional cycles of [^177^Lu]Lu-PSMA-617 RPT had significantly higher PSA response rates than did the 21 patients who were treated with a new line of systemic therapy (8).

Nevertheless, because of the retrospective nature and small sample sizes of these studies as well as the wide range of PSA response and adverse event rates, prospective data are needed to better inform treatment-making decisions for patients with mCRPC whose disease recurs after [^177^Lu]Lu-PSMA-617 RPT.

In this prospective phase 2 trial, titled RE-LuPSMA, we aim to evaluate the efficacy and safety of rechallenge therapy with [^177^Lu]Lu-PSMA-617 in patients with disease progression after responding well to a previous regimen of [^177^Lu]Lu-PSMA-617.

## MATERIALS AND METHODS

### Study Design

RE-LuPSMA is an investigator-initiated, open-label, single-arm, single-center, phase 2 trial conducted at the UCLA. This trial is financially supported by Novartis, is being conducted under Investigational New Drug application 169476, and was approved by the UCLA institutional review board (IRB23-001509). The trial is registered at ClinicalTrials.gov (NCT06288113).

**FIGURE 1.**
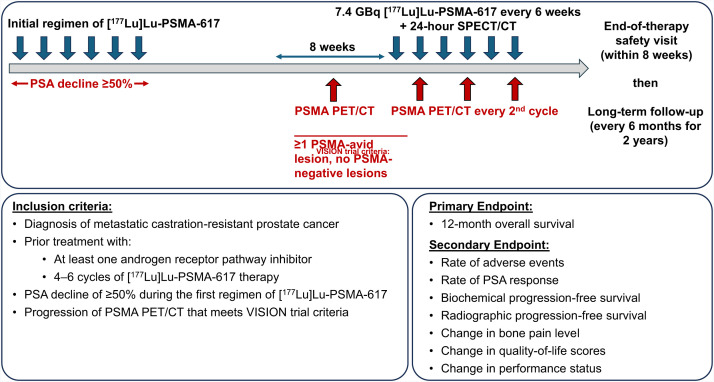
Study design.

**FIGURE 2.**
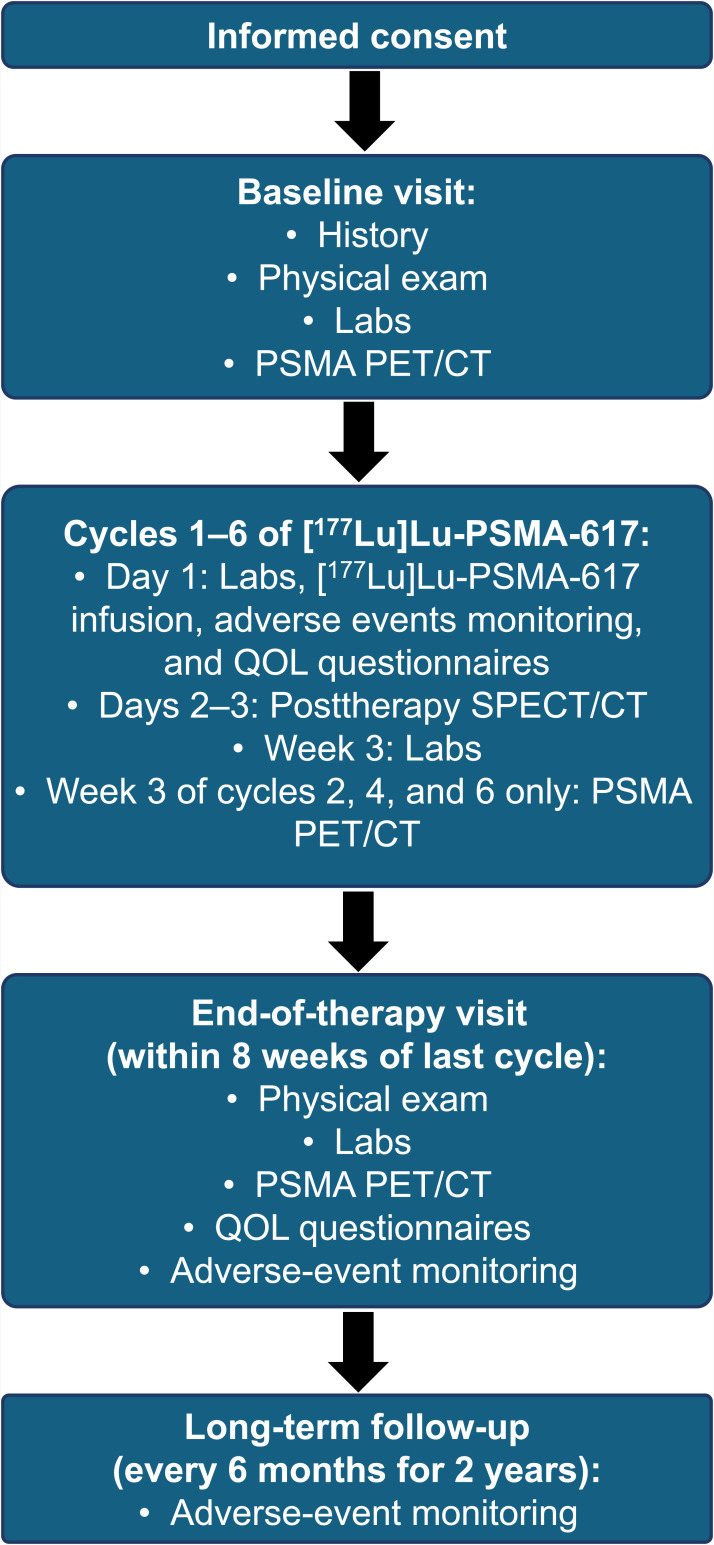
Patient flow chart. QOL = quality of life.

The study was powered to determine the 12-mo OS rate of patients with mCRPC who previously had a favorable response to a first regimen of [^177^Lu]Lu-PSMA-617 and are treated with rechallenge therapy (for a maximum of 6 additional cycles). We hypothesized a 12-mo OS rate of 71%, compared with a null hypothesis of an OS rate of 50% with no rechallenge therapy. The planned sample size is 40 subjects to achieve 80% power. The planned study duration is 45 mo. The study design is presented in Figure 1, and the patient flowchart is provided in Figure 2. The full study protocol is provided in the supplemental materials, available at http://jnm.snmjournals.org.

### Endpoints

The primary endpoint is the 12-mo OS rate, measured from cycle 1 day 1 of [^177^Lu]Lu-PSMA-617 rechallenge therapy, which will be estimated using the Kaplan–Meier method with a 95% CI. A 1-sample log-rank test (as used in the power calculation) will be applied to assess whether the observed OS rate exceeds the null rate of 50%, derived from the standard care arm of the phase 3 VISION trial (1).

The secondary endpoints are as follows:

- Overall and grade 3 or greater adverse-event rates, assessed using Common Terminology Criteria for Adverse Events version 5.0 through study completion.
- PSA response rate (response defined as a decline of ≥50% on 2 PSA levels measured at least 3 wk apart) during [^177^Lu]Lu-PSMA-617 rechallenge therapy.
- Biochemical PFS (defined by Prostate Cancer Working Group 3 guidelines as an increase of 25% and 2 ng/mL above the nadir, confirmed by a second value ≥ 3 wk later (11)) through rechallenge study completion.
- OS rate measured from the start of the first regimen of [^177^Lu]Lu-PSMA-617 RPT (cycle 1 day 1) through rechallenge study completion.
- OS rate measured from the end of the first regimen of [^177^Lu]Lu-PSMA-617 RPT (day 1 of the final cycle) through rechallenge study completion.
- Radiographic PFS through study completion, defined as progressive disease on prostate-specific membrane antigen (PSMA) PET/CT (Table 1) (12) or SPECT/CT Table 2 (13), assessed using response assessment criteria.
- Proportion of patients who initially had bone pain of at least 4/10 in severity who achieved a pain response (i.e., improvement of at least 2 points from baseline without an overall increase in opiate use) through rechallenge therapy completion.
- Median change in the Functional Assessment of Cancer Therapy–Radionuclide Therapy score (14) through study completion.
- Median change in the Brief Pain Inventory–Short Form score through rechallenge therapy completion.
- Median change in performance status, measured using the Eastern Cooperative Oncology Group performance score, through rechallenge therapy completion.

**TABLE 1.** PSMA PET/CT Response Assessment Criteria

Response	Definition
Complete response	Absence of any PSMA uptake and no new PSMA-negative tumors
Partial response	>30% decrease in PSMA-VOL with no new PSMA-positive or -negative tumors
Stable disease	<30% decrease in PSMA-VOL (with or without new PSMA-positive or -negative tumors), ≥30% decrease in PSMA-VOL with new PSMA-positive or -negative tumors, <20% increase in PSMA-VOL (with or without new PSMA-positive or -negative tumors), or ≥20% increase in PSMA-VOL without new PSMA-positive or -negative tumors
Progressive disease	>30% increase in PSMA-VOL (with or without sites of PSMA-positive or -negative tumors)

PSMA-VOL = total volume of all PSMA-positive tumors.

Adapted from (12).

**TABLE 2.** SPECT/CT Response Assessment Criteria

Response	Definition
Complete response	Absence of any PSMA uptake and no new PSMA-negative tumors
Partial response	>30% decrease in PSMA-VOL with no new PSMA-positive or -negative tumors
Stable disease	≤30% change in PSMA-VOL and no new PSMA-positive or -negative tumors
Progressive disease	>30% increase in PSMA-VOL (with or without new PSMA-positive tumors) or PSMA-negative tumors.

PSMA-VOL = total volume of all PSMA-positive tumors.

Adapted from (13).

### Eligibility Criteria

To qualify for enrollment, patients must meet all of the following inclusion criteria:

- Age ≥ 18 y.
- Diagnosis of mCRPC.
- Prior regimen of chemotherapy for mCRPC allowed but not required.
- Previous treatment with at least 1 ARPI.
- Previous completion of 4–6 cycles of [^177^Lu]Lu-PSMA-617.
- Demonstration of favorable response to the first regimen of [^177^Lu]Lu-PSMA-617, defined as a decline of at least ≥ 50% in PSA level.
- Meets PSMA PET/CT VISION trial criteria (1): at least 1 lesion with PSMA uptake higher than that of the liver, no soft-tissue lesions larger than 1 cm with PSMA uptake less than or equal to the liver, PSMA PET/CT must be performed within 8 wk of the planned first cycle of rechallenge therapy and at least 6 wk after completion of the first regimen of [^177^Lu]Lu-PSMA-617.
- Eastern Cooperative Oncology Group performance score of 0–2.
- Sufficient bone marrow capacity (defined as white blood cells ≥ 2,500/μL, platelets ≥ 100,000/μL, hemoglobin ≥ 9.0 g/dL, and absolute neutrophil count ≥ 1,500/μL).
- Abilities to understand and sign an approved informed-consent form and comply with all protocol requirements.

Also, HIV-infected patients who have well-controlled HIV and a low risk of AIDS-related outcomes are eligible for inclusion.

Patients are not eligible for study inclusion if they have received myelosuppressive therapy or other RPT within the past 6 wk (including docetaxel, cabazitaxel, ^89^Sr, ^153^Sm, ^186^Re, ^188^Re, and ^223^Ra) or have an estimated glomerular filtration rate of less than 50 mL/min/1.73 m^2^.

### Investigational Plan

Patients will receive intravenous injections of 7.4 GBq (±10%) of [^177^Lu]Lu-PSMA-617 once every 6 wk (±1 wk) for a maximum of 6 rechallenge cycles. [^177^Lu]Lu-PSMA-617 will be supplied by Novartis in vials for injection. Further details regarding dosage, packaging, labeling, and storage, can be found in sections 4.2.1–4.2.4 of the study protocol.

Patients will undergo hematology, chemistry, and PSA testing twice per cycle. The first time will be either on day 1 or within the 7 days preceding day 1. The second time will be during week 3.

Single-time-point SPECT/CT dosimetry protocol will be performed every cycle. Patients will be imaged with posttreatment quantitative SPECT/CT scans 24 h after administration of [^177^Lu]Lu-PSMA-617 (allowed window, 24–72 h) (15) using an Intevo T6 Dual Detector SPECT scanner with 6-slice CT (Siemens Healthineers). SPECT/CT scans will extend from midthigh to vertex with 3 bed positions. Dosimetry will be performed by using MIM (MIM Software Inc.). More details can be found in section 6.8 of the study protocol. SPECT/CT scans will be used to determine the maximum number of cycles each subject may receive without exceeding the kidney dose threshold (biologically effective dose [BED] of 39 Gy) and to evaluate response to treatment using response assessment criteria.

PSMA PET/CT scans will be performed during week 3 of cycles 2, 4, and 6 using Biograph 64 and mCT scanners (Siemens Healthineers). A single 185-MBq dose (range, 111–2,830 MBq) of [^68^Ga]Ga-PSMA-11 will be administered per PET scan as a bolus intravenous injection. After 50–100 min of uptake, patients will undergo a whole-body (skull to midthighs) PET/CT scan. PET emission scans will be corrected for decay, dead time, random events, and scatter. PET images will be corrected for attenuation using segmented attenuation data of the low-dose CT scan. More details are provided in section 6.7 of the study protocol.

Table 3 details how treatment-related adverse events will be graded and the appropriate management recommendations for each adverse event.

**TABLE 3.** Management of Treatment-Related Adverse Events

Adverse event and severity	Management recommendation
Myelosuppression (anemia, thrombocytopenia, leukopenia, or neutropenia)	
Grade 2	[^177^Lu]Lu-PSMA-617 can be given as clinically indicated.
Grade ≥3	Withhold [^177^Lu]Lu-PSMA-617 until improvement to grade 1 or return to baseline, then reduce dose by 20% to 5.9 GBq.
Renal toxicity	
Grade 2 with eGFR < 30 mL/min/1.73 m^2^	Withhold [^177^Lu]Lu-PSMA-617 until improvement or return to baseline.
≥40% increase in serum creatinine and >40% decrease in eGFR compared with baseline	Withhold [^177^Lu]Lu-PSMA-617 until improvement or return to baseline, then reduce dose by 20% to 5.9 GBq.
Grade ≥3	Permanently discontinue [^177^Lu]Lu-PSMA-617.
Dry mouth	
Grade 2	Withhold [^177^Lu]Lu-PSMA-617 until improvement or return to baseline; consider reducing dose by 20% to 5.9 GBq.
Grade 3	Withhold [^177^Lu]Lu-PSMA-617 until improvement or return to baseline, then reduce dose by 20% to 5.9 GBq.
Gastrointestinal toxicity	
Grade ≥3 that is not amenable to medical intervention	Withhold [^177^Lu]Lu-PSMA-617 until resolved to grade 2 or return to baseline, then reduce dose by 20% to 5.9 GBq.
Fatigue	
Grade ≥3	Withhold [^177^Lu]Lu-PSMA-617 until resolved to grade 2 or return to baseline.
Electrolyte or metabolic abnormalities	
Grade ≥2	Withhold [^177^Lu]Lu-PSMA-617 until improvement to grade 1 or return to baseline.
AST or ALT elevation >5 times ULN in absence of liver metastases	Permanently discontinue [^177^Lu]Lu-PSMA-617.
Other nonhematologic toxicity	
Any unacceptable toxicity	Permanently discontinue [^177^Lu]Lu-PSMA-617.
Any serious adverse event that requires treatment delay >4 wk	Permanently discontinue [^177^Lu]Lu-PSMA-617.
Any persistent and intolerable grade ≥2 adverse event	Permanently discontinue [^177^Lu]Lu-PSMA-617.

eGFR = estimated glomerular filtration rate; AST = aspartate transaminase; ALT = alanine transaminase; ULN = upper limit of normal.

During this trial, the only concomitant medications that patients are allowed to continue are androgen-deprivation therapy (e.g., leuprolide, relugolix), first-generation ARPIs (e.g., bicalutamide), local external beam radiotherapy, and concomitant prostate cancer therapies that were administered during the first regimen of [^177^Lu]Lu-PSMA-617 and continued afterward. Any other treatments for prostate cancer must be discontinued before the start of the trial. Second-generation ARPIs (e.g., abiraterone, enzalutamide, darolutamide, apalutamide) do not require a washout period. Chemotherapy agents (e.g., docetaxel, cabazitaxel) require a 6-wk washout period. RPTs (e.g., ^89^Sr, ^153^Sm, ^223^Ra) require a 6-wk washout period. Sipuleucel-T, olaparib, rucaparib, and pembrolizumab require a 4-wk washout period.

### Study Duration

Patients will be treated with up to 6 cycles of [^177^Lu]Lu-PSMA-617 rechallenge therapy. Treatment will be discontinued if there is evidence of biochemical (PSA increase of >25% and 2 ng/mL above nadir) and radiographic (progressive disease on PSMA PET/CT or SPECT/CT) progression, withdrawal of consent, noncompliance, unacceptable toxicity, or evidence that the patient is no longer benefiting or at the discretion of the investigator or sponsor.

**FIGURE 3.**
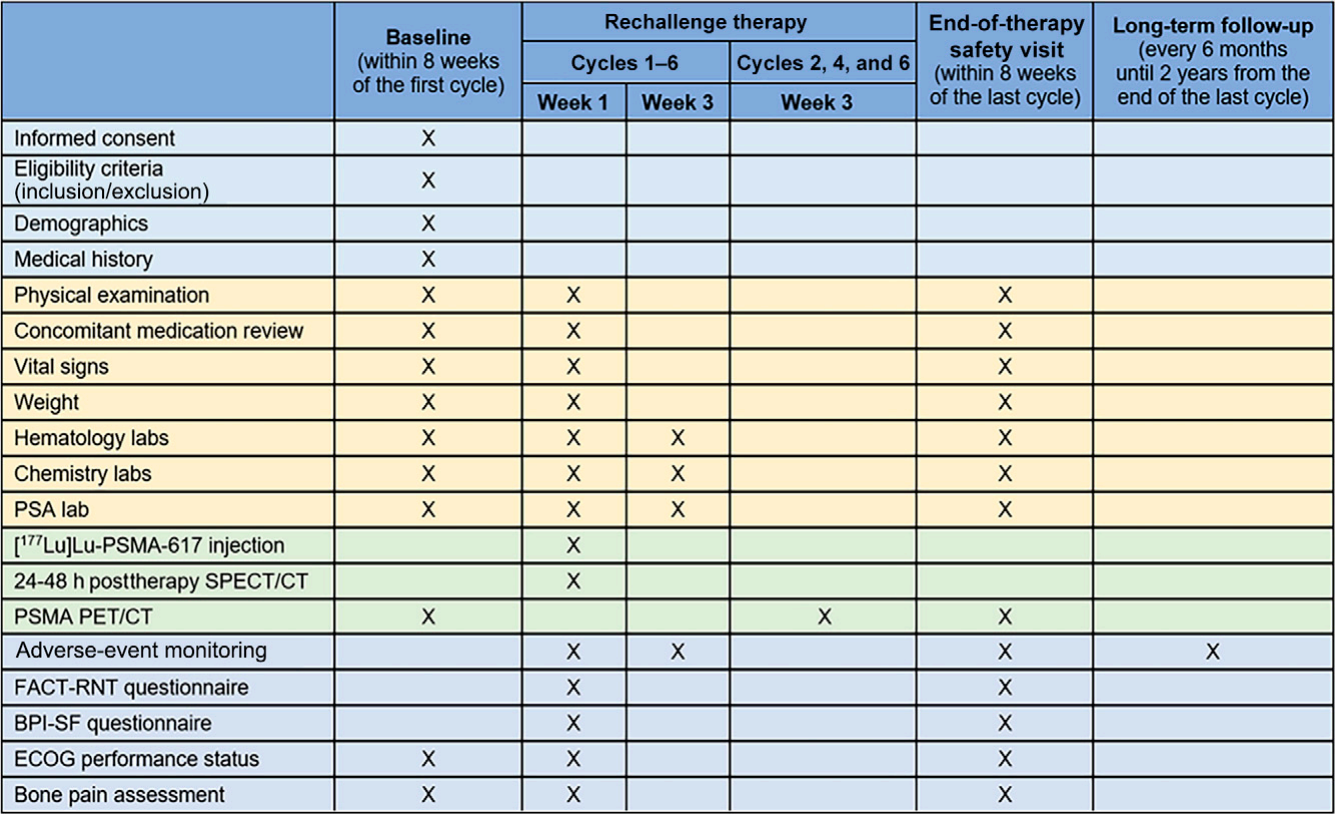
Schedule of assessments. BPI-SF = Brief Pain Inventory–Short Form; ECOG = Eastern Cooperative Oncology Group; FACT-RNT = Functional Assessment of Cancer Therapy–Radionuclide Therapy.

The duration of subject participation will be from the time informed consent is provided through the 24 mo following completion of the final cycle of rechallenge therapy. The planned study duration is 45 mo. Study enrollment began August 2024, with an anticipated trial completion date of May 2028. The full schedule of visits and assessments is provided in Figure 3.

### Sample Size Determination and Stopping Rule

The primary endpoint of the study is the 12-mo OS rate starting from cycle 1 day 1 of rechallenge therapy. We hypothesize that patients retreated with [^177^Lu]Lu-PSMA-617 will have a 12-mo OS rate of 71% compared with a null hypothesis of 50% with no rechallenge therapy, based on the standard care group of the phase 3 VISION trial (1). A 2-sided, 1-sample log-rank test was used to estimate power. Assuming survival times follow an exponential distribution, a total of 40 subjects will achieve 80% power to detect a difference between a rechallenge group (71%) and a historic control group (50%), using a significance level of 0.05 (2-sided) and a follow-up duration of 24 mo after the last subject is enrolled. The complete statistical analysis plan is provided in section 11 of the study protocol.

The study incorporates a stopping rule mandating termination of the trial if treatment-related serious adverse events occur in 5 of the first 20 patients. If 4 or fewer such events occur among the first 20 patients, the trial can continue to completion. This rule was informed by an expected event rate of approximately 8% rate, as reported in prior retrospective series (6–10), using a 1-sample binomial test (1-sided α = 0.05).

## DISCUSSION

This represents one of the first prospective phase 2 trials of rechallenge therapy for patients with mCRPC whose disease recurs after a favorable response to an initial regimen of [^177^Lu]Lu-PSMA-617 and who may respond favorably if rechallenged. This trial aims to establish both the efficacy of rechallenge therapy, using 12-mo OS as the primary endpoint, as well as the safety of such therapy, using the rate of adverse events as well as multiple quality-of-life measures as secondary endpoints.

The inclusion criteria specify that patients must have had a PSA response, defined as a decrease of at least 50%, during the initial regimen. Patients must also have completed at least 4 cycles of [^177^Lu]Lu-PSMA-617 during the initial regimen, as completing a shorter course likely signaled that the treatment was stopped because of significant adverse effects or disease progression. These 2 inclusion criteria are supported by several retrospective studies that showed favorable outcomes for rechallenge therapy (7–9).

The 12-mo OS rate was chosen to be the primary endpoint because the VISION trial showed such a dramatic OS benefit for [^177^Lu]Lu-PSMA-617 RPT (hazard ratio, 0.62) (1). We hypothesize that the OS benefit of rechallenge therapy will be similar to that observed in the original VISION trial (i.e., approximately 40% mortality reduction). Even though patients may not have as strong of a response to the second regimen of [^177^Lu]Lu-PSMA-617 as they did to the initial regimen, this may be balanced by the fact that this trial is only enrolling patients who responded well to the initial regimen. Using the 12-mo OS rate of 50% from the VISION trial standard-of-care group as a historical benchmark and assuming a mortality reduction of 40% with rechallenge therapy, we hypothesize that we will observe a 12-mo OS rate of greater than 70%. This is the value that was used for the power analysis of the primary endpoint. We decided against using a retrospective cohort of rechallenge therapy as our comparison cohort because of the limitations in sample size, selection bias, and the retrospective nature of data collection.

Prior retrospective data have not established the ideal number of cycles for rechallenge therapy. Reports range from 2 additional cycles to as many as 3 separate courses of 1–6 cycles per course (6–10). We hope to determine how a repeat course of up to 6 additional cycles performs in this patient population.

At the request of the FDA, our study protocol includes dosimetric analyses using a single-time-point SPECT/CT scan after each infusion of [^177^Lu]Lu-PSMA-617. The FDA raised the issue that, between the 2 regimens of [^177^Lu]Lu-PSMA-617 as well as any prior external beam radiotherapy, patients may exceed the maximum dose constraint for the kidneys. This constraint has been established as a BED of 39 Gy, using data from peptide receptor RPT (16). The protocol was modified to specify that if the calculated kidney BED exceeds 39 Gy, the total number of cycles must be adapted or limited to remain under the 39-Gy BED limit.

When the trial began, there was an additional inclusion criterion that patients must have had at least 1 course of prior chemotherapy, consistent with FDA indications for [^177^Lu]Lu-PSMA-617 RPT. This was removed in a protocol amendment after results from the PSMAfore trial showed that chemotherapy-naïve patients had a radiographic PFS benefit with [^177^Lu]Lu-PSMA-617 compared with a change in ARPI therapy (17), which led to FDA approval of [^177^Lu]Lu-PSMA-617 in chemotherapy-naïve patients as well.

Since this study began, we have considered several exploratory analyses. The first includes studying how the presence of concomitant medications affects OS and PSA response. This question has not been addressed by existing retrospective data. We plan to compare patients receiving any concomitant medication with patients receiving no concomitant medications using log-rank analysis. We also plan to compare patients receiving androgen-deprivation therapy or first-generation ARPIs with those who continued second-generation ARPIs using log-rank analyses. The second exploratory analysis includes evaluating whether the length of time between the initial [^177^Lu]Lu-PSMA-617 course and subsequent PSA progression affects OS and PSA response rates. We plan to compare patients with time intervals less than 12 mo versus time intervals greater than or equal to 12 mo using log-rank analysis.

## CONCLUSION

The RE-LuPSMA trial is a prospective phase 2 trial studying the efficacy and safety of [^177^Lu]Lu-PSMA-617 rechallenge therapy. This has the potential to establish an alternative therapeutic option for patients with progressive mCRPC after a favorable response to an initial regimen of [^177^Lu]Lu-PSMA-617.

## DISCLOSURE

RE-LuPSMA is an investigator-initiated trial financially supported by Novartis. The study protocol was developed by the investigators without any industrial or other third-party involvement. John Nikitas is a recipient of the Christiaan W. Schiepers Theranostics Fellowship award. Jeremie Calais reports grants from the Prostate Cancer Foundation (2020 Young Investigator Award 20YOUN05), the Society of Nuclear Medicine and Molecular Imaging (2019 Molecular Imaging Research Grant for Junior Academic Faculty), the Philippe Foundation Inc., and the ARC Foundation (International Mobility Award SAE20160604150) and grants of support paid to his institution from Lantheus, Novartis, and POINT Biopharma. He also reports consulting activities for Advanced Accelerator Applications, Amgen, Astellas, Bayer, Blue Earth Diagnostics, Curium Pharma, DS Pharma, Fibrogen, GE HealthCare, Isoray, IBA RadioPharma, Janssen Pharmaceuticals, Monrol, Lightpoint Medical, Lantheus, Novartis, Pfizer, POINT Biopharma, Progenics, Radiomedix, Sanofi, Siemens-Varian, SOFIE Biosciences, and Telix Pharmaceuticals, outside of the submitted work. Adrien Holzgreve reports funding by the Deutsche Forschungsgemeinschaft (German Research Foundation 545058105) and compensation for scientific consulting from ABX advanced biochemical compounds, outside of the submitted work. Lena Unterrainer reports support from the Munich Clinician Scientist Program and the Bavarian Cancer Research Center. Johannes Czernin is the recipient of a grant from the Prostate Cancer Foundation (2017 and 2019 Challenge Awards 17CHAL02 and 19CHAL09) and the Jonsson Comprehensive Cancer Center NIH–National Cancer Institute Cancer Center Support Grant (P30 CA016042), a founder of SOFIE Biosciences and Trethera Corp., and a scientific advisory board member of Aktis Oncology. No other potential conflict of interest relevant to this article was reported.
